# The Impact of Proton Pump Inhibitors on the Development of Gastric Neoplastic Lesions in Patients With Autoimmune Atrophic Gastritis

**DOI:** 10.3389/fimmu.2022.910077

**Published:** 2022-07-22

**Authors:** Emanuele Dilaghi, Mario Bellisario, Gianluca Esposito, Marilia Carabotti, Bruno Annibale, Edith Lahner

**Affiliations:** Department of Medical-Surgical Sciences and Translational Medicine, Sant’Andrea Hospital, Sapienza University of Rome, Rome, Italy

**Keywords:** autoimmune atrophic gastritis, gastric cancer, proton pump inhibitor (PPI), gastric neoplastic lesion, gastric autoimmunity

## Abstract

**Introduction:**

Proton pump inhibitors (PPIs) have been widely prescribed as a primary treatment for acid-related disorders. A large body of literature reported several adverse outcomes due to PPI therapy, including an increased risk of gastric cancer (GC). Autoimmune atrophic gastritis (AAG) is a chronic inflammatory disorder affecting the oxyntic mucosa, leading to mucosal atrophy, intestinal metaplasia, and reduced gastric acid secretion, up to the possible development of dysplasia and intestinal-type GC. Whether PPI use may increase the GC risk in AAG patients has not yet been investigated. We conducted a case–control study in AAG patients to assess the association between the PPI use before AAG diagnosis and the development of GC at follow-up (FU).

**Materials and Methods:**

Patients were included from a prospective cohort of AAG patients (diagnosed 1992–2021) in a referral center for gastric autoimmunity; all patients adhered to an endoscopic–histological FU program according to Management of precancerous conditions and lesions in the stomach (MAPS) I/II (management of epithelial precancerous conditions) guidelines. At diagnosis, clinical/biochemical data and PPI use before AAG diagnosis (withdrawn at the time of diagnosis), for at least 12 months, were evaluated. Patients who developed gastric neoplastic lesions (GNLs) at FU were considered as cases; patients without a diagnosis of GNLs at FU were considered as controls. At a total FU of 2.3 years (1–13), 35 cases were identified, and controls were matched 2:1 by age ( ± 3 years), gender, and years of FU (n=70); therefore, a total of n=105 patients were included in the study.

**Results:**

The proportion of PPI users before AAG diagnosis was significantly higher in cases than in controls (54.3% vs. 18.6%, *p*<0.001). At logistic regression, considering as a dependent variable the development of GNLs at FU, a positive association was shown for PPI use before AAG diagnosis (OR 9.6, 95%CI 2.3–40.3), while other independent variables as the use of antiplatelets/anticoagulants (OR 2.8, 95%CI 0.7–12.0), age ≥ 50 years (OR 2.0, 95%CI 0.2–18.1), 1st-degree family history for GC (OR 2.4, 95%CI 0.4–15.2), and smoking habit (OR 0.4, 95%CI 0.1–2.1) were not associated.

**Conclusions:**

PPI use before the diagnosis of AAG appears to considerably increase the risk of subsequent GNL development. Considering the common misuse of PPIs, physicians should regularly reevaluate the appropriateness of ongoing PPI therapy, in particular in patients with a clinical suspicion of or already diagnosed AAG.

## Introduction

Proton pump inhibitors (PPIs) are widely used all over the world. First introduced in the late 1980s, they are widely prescribed as a first-line treatment in patients affected by acid-related disorders, such as gastroesophageal reflux disease (GERD), erosive disease, peptic strictures, and Barrett’s esophagus. In addition, PPIs are used for preventing gastrointestinal bleeding while on nonsteroidal anti-inflammatory drugs, in eosinophilic esophagitis, and non-ulcer dyspepsia (1, 2), successfully relieving symptoms and further complications in many cases. Available PPIs include esomeprazole, omeprazole, pantoprazole, dexlansoprazole, lansoprazole, and rabeprazole. They inhibit active parietal cell acid secretion acting by the irreversible inhibition of H^+^/K^+^ ATPase, the proton pump present in the gastric parietal cells, responsible for acid production. Since their introduction, remarkable evidence arose on possible associations between PPI use and adverse events, including the role of PPIs in the development of gastric cancer (GC) (3). Recently, in 2021, the role of PPI was investigated as a risk factor for GC, in a large population-based cohort of study (4), showing that the use of PPIs was associated with a 45% increased risk of GC compared with the use of Histamine-2 receptor antagonists (H2RAs). Similarly, in the same year, another large population-based cohort study (5), showed how PPI use was associated with a 2.37-fold increase in the general population. The association between PPIs and GC is biologically plausible and may be mediated by several different factors: PPIs, by inhibiting acid production, cause hypergastrinemia. Gastrin, secreted by antral G-cells, is considered a potent growth factor that may lead to enterochromaffin-like (ECL) cell hyperplasia (6). Second, non-acid gastric pH may contribute to gastric dysbiosis with an eventual overgrowth of strains promoting carcinogenesis (7, 8). Third, chronic suppression of acid secretion by PPIs may accelerate or induce the progression of gastric corpus atrophy (9).

Autoimmune atrophic gastritis (AAG), a chronic inflammatory disorder affecting the oxyntic mucosa, leads to progressive mucosal atrophy, reduced gastric acid secretion, and eventually to intestinal metaplasia (10), up to the possible development of epithelial lesions such as dysplasia and intestinal-type GC. AAG, which manifests itself most commonly with anemia or long-standing dyspepsia, is considered a condition at increased risk for GC (10). Whether PPIs may increase the risk of GC in AAG patients has not yet been investigated. This study aimed to investigate the relationship between PPI use before AAG diagnosis and the subsequent development of gastric neoplastic lesions (GNLs) at follow-up (FU) in a prospective cohort of AAG patients.

## Materials and Methods

This article was drafted according to the Strengthening the Reporting of Observational Studies in Epidemiology (STROBE) guidelines (**Supplementary Table 1**) to ensure the quality of reporting (11).

### Study Design and Participants

A case–control study was conducted, including AAG patients from a prospective cohort that adhered to an endoscopic–histological FU program (12) scheduled every 4 years before 2011 and then every 3 years according to the guidelines (13, 14). In the case of symptoms not reported during previous evaluations, the worsening of the same, or the onset of anemia, a gastroscopy was anticipated. Cases and controls were selected from a prospective cohort of 560 patients with AAG (diagnosed 1992–2021, 69.4% female, median age 59.7 [18–92] years) in a teaching hospital and referral center for gastric autoimmunity. Cases were defined as those patients with AAG who developed GNLs (such as low- and high-grade dysplasia, and intestinal-type GC) at FU and controls as those patients with AAG who did not develop any GNLs at FU.

At diagnosis, clinical and hematological–serological data were assessed by a structured questionnaire. Prior use of PPIs, for at least 12 months (15) (regardless of the PPI class), was recorded by a specific item in the structured questionnaire. These drugs were withdrawn at the time of AAG diagnosis due to the already-reduced or absent gastric acid secretion as a consequence of the atrophy of corpus oxyntic glands in AAG. Patients were also advised to avoid the use of these drugs in the future.

**Figure 1:**
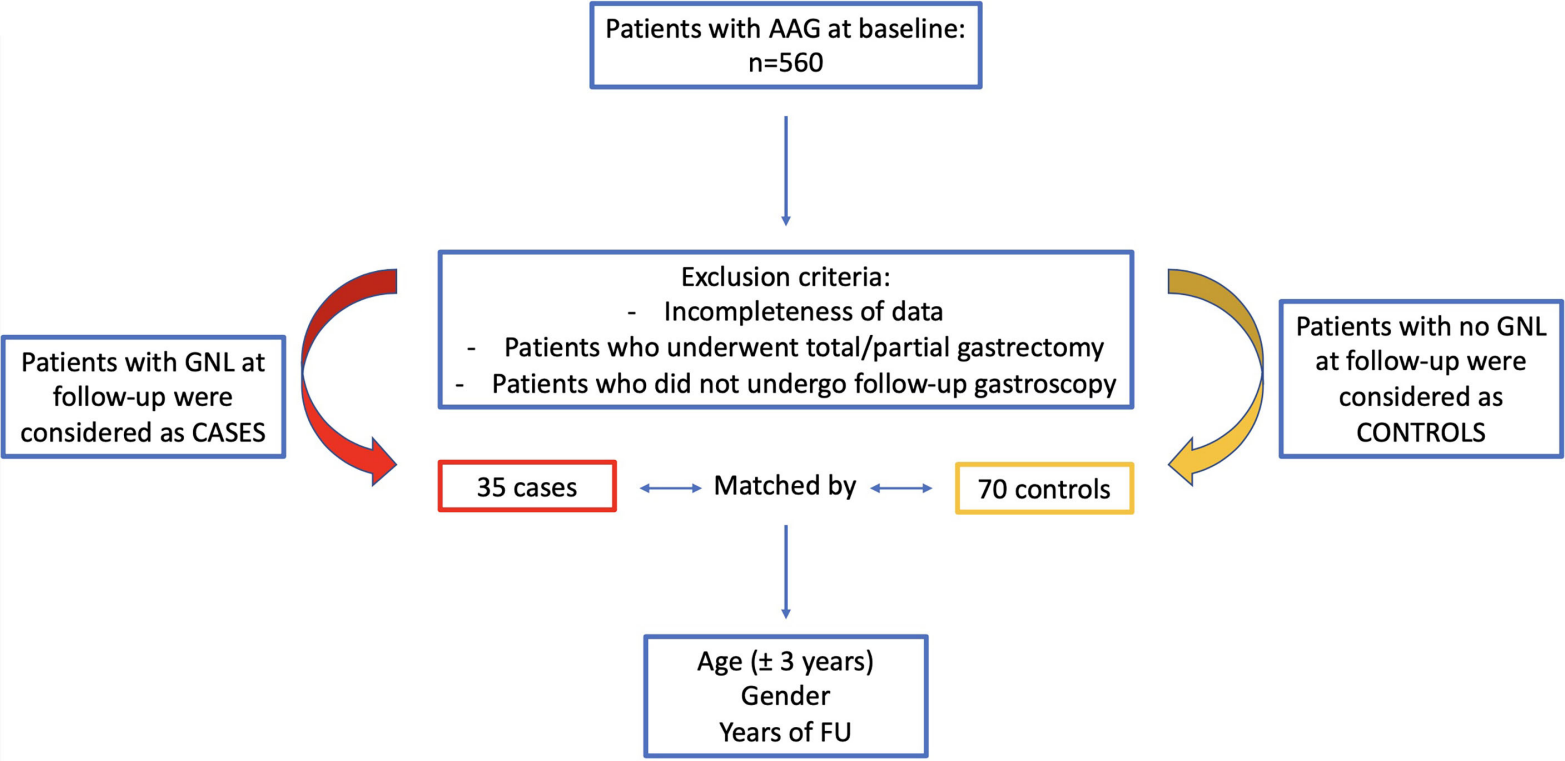
Flowchart of the study population of patients with autoimmune atrophic gastritis included in the case–control study.

Inclusion criteria were as follows: the presence of AAG, confirmed with the histopathological assessment of gastric biopsies; completeness of personal, clinical, hematological–serological, endoscopic, and histopathological data of gastric biopsies; and eventual epithelial GNLs. Exclusion criteria were as follows: age <18 years; incompleteness or unavailability of previously reported data, and/or inadequacy of gastric biopsies; patients who underwent partial or total gastrectomy in case of a neoplastic gastric lesion at baseline; and patients who did not undergo FU gastroscopy. Anemia and *Helicobacter pylori* (Hp) infection were diagnosed as previously reported (16). The treatment of patients with active Hp infection (n=18) was performed as previously reported (17); all patients were successfully cured of Hp before FU. From the prospective cohort of AAG patients, a total of 105 patients were included: cases included 35 patients (57.1% females, median age 67.6 [44–84] years) who at a median FU of 2.3 years (range 1–13 years) developed GNLs. **Table 1** gives the main clinical features of cases. Controls included 70 patients that were matched (2:1) to cases by age ( ± 3 years), sex, and FU. Controls were selected considering age- and sex-matched AAG patients with an FU period (from the time of AAG diagnosis to the last available surveillance endoscopy) matched to the FU period of cases but without having developed a GNL. **Figure 1** summarizes the flowchart of the study population.

**Table 1:** Baseline characteristics of 35 cases with autoimmune atrophic gastritis and gastric neoplastic lesions.

	Number (%)
Females	20 (57.1)
Median age, years, median (range) Patients >50 years of age	67.6 (44–84) 32 (91.4)
Overall follow-up, years, median (range) Prior use of PPIs	2.3 (0–13) 19 (54.3)
Body mass index ≥25 kg/m²	17 (48.6)
Smoking habit	4 (11.4)
First-degree family history for gastric cancer	3 (8.6)
Dyspepsia Use of antiplatelet or anticoagulant drugs	17 (48.6) 14 (40.0)
Positivity toward parietal cell antibodies	24 (68.6)
Iron deficiency anemia	4 (11.4)
Pernicious anemia	13 (37.1)
Severe corpus atrophy	13 (37.1)
Presence of corpus intestinal metaplasia	26 (74.3)

Data are expressed as number (%) when not otherwise indicated.

Informed consent was provided by all participants, and approval of the local ethical committee was achieved (No. 7022/2020).

### Endoscopic Procedures and Biopsies

Gastroscopies were performed using Olympus scopes (GIF-H180, GIF-H185, or GIF-HQ190) by endoscopists with the use of pharyngeal anesthesia (xylocaine spray puffs) and/or conscious sedation (midazolam). Gastroscopy was conducted with the use of HR-white light endoscopy, and biopsies were performed according to the updated Sydney system protocol (18): two biopsies of the antrum, one of the *incisura angularis*, and two of the corpus. Biopsies were then sent for histopathological evaluation in two separate vials, one from the antrum (two biopsies of antral mucosa plus one of the *incisura*) and one from the corporal mucosa. Additional biopsies were taken if other lesions were present and sent for histopathological evaluation.

### Histopathological Assessment

AAG was defined as the decrease or disappearance of the oxyntic glands that could be replaced by fibrosis or most frequently by pseudo-pyloric or intestinal metaplasia, associated to ECL hyperplasia. Gastric mucosal atrophy was restricted to the corpus and fundus glands of the stomach, sparing the antrum (10, 19, 20). The presence of an active Hp infection was detected by modified Giemsa staining.

Based on the morphological histopathological assessment, corpus mucosa IM was defined as the substitution of the normal oxyntic glands with intestinalized glands, according to the updated Sydney system (18).

### Statistical Analysis

Data were expressed as median (range) and/or number/total (percentage, %). Differences between cases and controls concerning clinical, serological, and histological variables were analyzed using the chi-squared test or Fisher’s exact test, as appropriate. Two-tailed *p*-values <0.05 were considered statistically significant. To assess eventual predictors associated with the development of GNLs, multivariate analyses based on logistic regression (stepwise and forward methods) were performed by using as dependent variable the GNL development at FU and as independent variables those cofactors that were significantly more or less frequent in patients with GNLs than in patients without GNLs at univariate analysis or that reasonably could have an association with the development of GNLs. Statistical analyses were performed by MedCalc^®^ Statistical Software version 19.6.4 (MedCalc Software Ltd, Ostend, Belgium; https://www.medcalc.org; 2021).

## Results

Patients with AAG who developed GNLs at FU were 35 (cases); 57.1% of patients were female gender and the median age was 67.6 (range 44–84) years. Cases were followed up for a median period of 2.3 (range 1–13) years, and the following GNLs were detected: 21 (60.0%) low-grade dysplasia, 3 (8.6%) high-grade dysplasia, and 11 (31.4%) intestinal-type GC. Regarding patients who presented low-grade dysplasia lesions at actual FU, none progressed to more invasive lesions, such as high-grade dysplasia or GC. The main features of the patients with GNLs are reported in **Supplementary Tables 2, 3**.

Controls were 70 AAG patients without any GNLs at FU, and they were matched considering the age ( ± 3 years) at diagnosis, gender, and time of FU; 60% of controls were female, and the median age was 67.7 (range 42–86) years. The comparison of the main features between cases and controls is given in **Table 2**.

**Table 2:** Comparison of main characteristics between patients with autoimmune atrophic gastritis with gastric neoplastic lesions (cases) and patients with autoimmune atrophic gastritis without gastric neoplastic lesions (controls).

	Cases n=35	Controls n=70	*p*-value
Females	20 (57.1)	42 (60.0)	NS (matched)
Median age, years, median (range) Age >50 years Prior use of PPIs	67.6 (44–84) 32 (91.4) 19 (54.3)	67.7 (42–86) 65 (92.9) 13 (18.6)	NS (matched) NS (matched) <0.001
Body mass index ≥25	17 (48.6)	26 (37.1)	0.297
Smoking habit	4 (11.4)	34 (48.6)	<0.001
First-degree family history for gastric cancer	3 (8.6)	7 (10.0)	1.000
Dyspepsia Use of antiplatelet or anticoagulant drugs	17 (48.6) 14 (40.0)	28 (40.0) 11 (15.7)	0.412 0.008
Iron deficiency anemia	4 (11.4)	13 (18.6)	0.412
Pernicious anemia	13 (37.1)	28 (40.0)	0.834
Severe corpus atrophy	13 (37.1)	27 (38.6)	1.000
Presence of corpus intestinal metaplasia	26 (74.3)	58 (82.9)	0.312

Data are expressed as number (%) when not otherwise indicated.NS, not significant.

The use of PPIs before the diagnosis of AAG was significantly more frequent in cases than in controls: 19 (54.3%) versus 13 (18.6%), (p<0.001).

Smoking habit was significantly more frequent in controls than in cases (48.6% vs. 11.4%, p<0.001). No significant difference was observed between cases and controls regarding the presence of severe corpus atrophy (37.1% vs. 38.6%, p=1.000).

**Figure 2:**
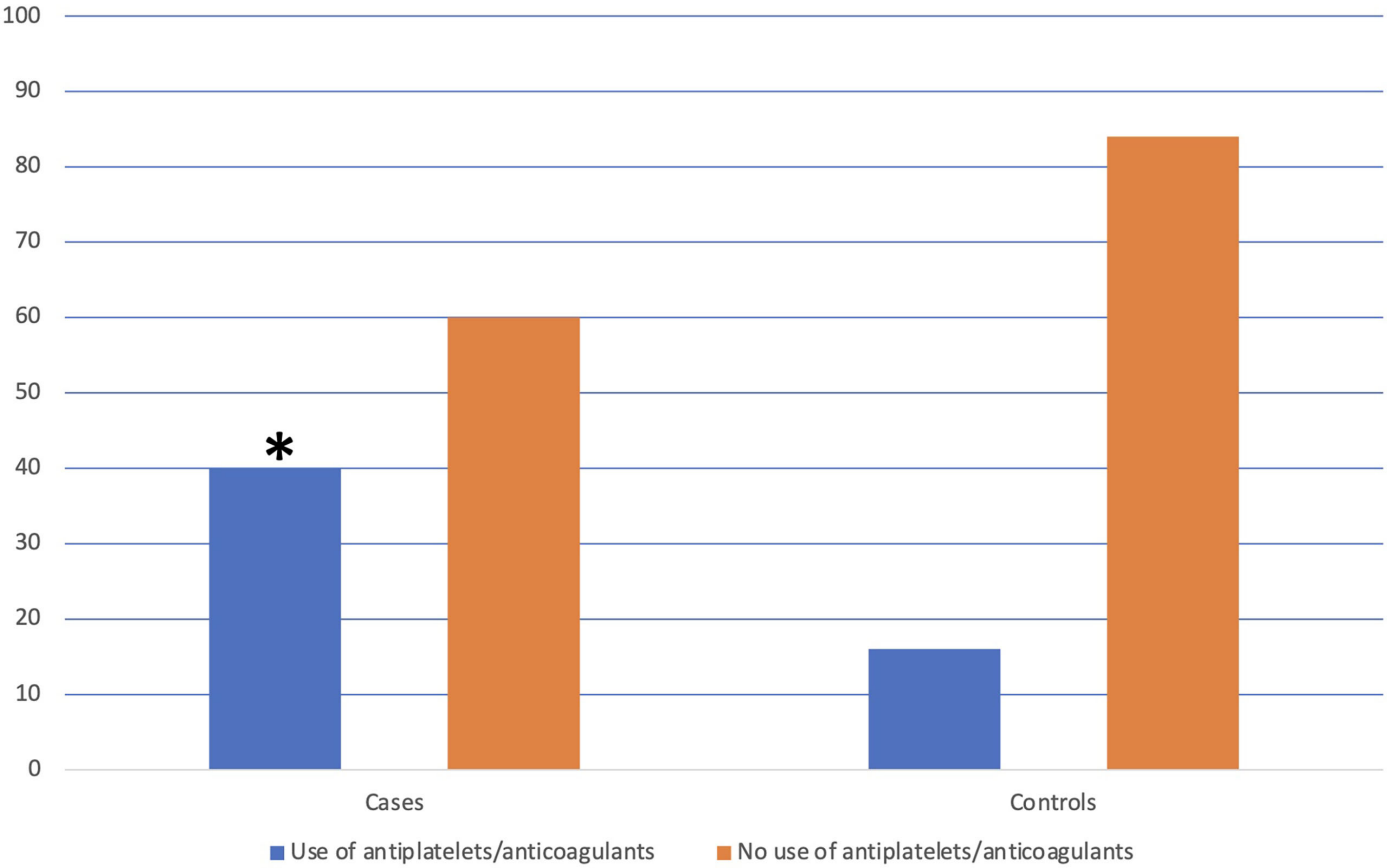
Distribution of the use of antiplatelets/anticoagulants in patients with and without the development of gastric neoplastic lesions. *The use of antiplatelets/anticoagulants was significantly more frequent in cases than in controls (40.0% vs. 15.7%, p=0.008).

The use of antiplatelet or anticoagulant drugs was reported in 14 (40.0%) cases vs. 11 (15.7%) controls; in particular, 3 cases reported the use of anticoagulant drugs, instead, none of the controls were on anticoagulant therapy. The use of antiplatelet or anticoagulant drugs was significantly more frequent in cases (p=0.008) (see **Figure 2**). At logistic regression, as reported in **Table 3**, a positive association with GNLs in AAG was shown for PPI use before AAG diagnosis with an odds ratio (OR) of 9.6 (95%CI 2.3–40.3) while the other independent variables as the use of antiplatelets/anticoagulants (OR 2.8, 95%CI 0.7–12.0), 1st-degree family history for GC (OR 2.4, 95%CI 0.4–15.2), age ≥50 years (OR 2.0, 95%CI 0.2–18.1), and smoking habit (OR 0.4, 95%CI 0.1–2.1) were not associated with the development of GNLs at FU.

**Table 3:** Features associated with the development of gastric neoplastic lesions in autoimmune atrophic gastritis (logistic regression).

	OR (95% CI)
**Prior PPI use**	
No	1.0
**Yes**	**9.6 (2.3-40.3)**
Use of antiplatelet or anticoagulant drugs	
No	1.0
Yes	2.8 (0.6-12.0)
First-degree family history for gastric cancer	
No	1.0
Yes	2.4 (0.4-15.2)
Age >50 years	
No	1.0
Yes	2.0 (0.2-18.1)
Smoking habit	
No	1.0
Yes	0.4 (0.1-2.1)

Bold values, to emphasize the result.

## Discussion

The current paper shows that in patients with AAG, the occurrence of GNLs was significantly related to the use of PPIs before the diagnosis of AAG. To the best of our knowledge, this is the first study focused on the association between the development of GNLs in patients with AAG. The association between GC and the use of PPIs has become a topic of considerable importance. In literature, several observational studies examined the association between PPIs and GC, all of which reported an increased risk with relative risks ranging between 1.1 and 3.6 (4, 5, 21–26). Salvo et al. (3), in 2020, performed an umbrella review to assess the associations between PPI use and adverse events. In addition to the significant association between PPIs and fractures, kidney disease, and infections (e.g., *Clostridioides difficile*), a significant pooled relationship was reported with GC development (OR 2.5, 95%CI 1.7–3.8). More recently, Seo SI et al. (5) analyzed two different Korean nationwide cohort databases and found that PPIs used for >30 days at a median FU of 4.3 years lead to a 2.4-fold GC risk when compared to non-PPI users, and GC incidence was associated with the duration of PPI use. Abrahami D et al. (4) investigated the GC risk associated with PPIs using a large population-based cohort from UK Clinical Practice Research Datalink showing that a PPI intake of median 5 years was associated with a 45% increased GC risk when compared to H_2_-blockers (HR 1.4, 95%CI 1.1–2.0). According to our findings, the use of PPIs before AAG diagnosis seems to considerably increase the probability to develop GNLs in subsequent years in AAG patients (OR 9.6, 95%CI 2.3–40.3). The mechanisms by which this may occur are not easy to explain, as the drug was withdrawn at the time of diagnosis of AAG. The long-term effect of previous PPI use in AAG may deteriorate the labile balance of these patients already characterized by an increased GC risk, eventually acting as a promotor of some of the mechanisms, such as the progression of gastric oxyntic atrophy and gastric dysbiosis (7–9).

To this day, a specific definition of the long-term use of PPIs is not available since different studies consider different timings concerning the purpose of that specific study: a systematic review (15) explored the definitions of long-term PPI use in literature and reported how the definition of long-term PPI treatment varied substantially between studies and was seldom rationalized. The authors reported how the definition of long-term use may change, considering a clinical instead of a research context. For a research context, >6 months have been proposed as a possible definition for long-term PPI use. In the current study, a PPI use of at least 12 months was considered that can be viewed as a pretty- confident definition of the long-term use of PPIs for the purpose of this study, also taking into consideration that the GC risk seems related to the duration of PPI assumption (27)

The use of antiplatelet/anticoagulants was significantly more frequent in cases than in controls (40.0% vs. 15.7%, p=0.008), but the multivariate analysis did not confirm the use of these drugs as an independent predictor for the development of GNLs. Probably, the frequent use of antiplatelets among cases may be explained as one possible reason why many of these patients were on PPI treatment due to the well-known gastrolesive and bleeding-promoting action of these drugs contrasted by the blockade of gastric acid secretion (27).

PPIs are a widely accepted treatment for dyspepsia (28). However, dyspeptic symptoms, such as epigastric pain, early satiety, and/or postprandial fullness, were reported in 35.5%, 10%, and/or 7% of AAG patients, respectively (29), and may be clinical clues leading to the AAG diagnosis. Moreover, dyspepsia may sometimes hide more relevant organic conditions, such as GC. In our study, dyspepsia was also reported in 48.6% and 40.0% cases and controls, respectively. Relieving dyspeptic symptoms with PPIs could delay the AAG diagnosis and accelerate the undergoing oxyntic atrophic process. Instead, patients with persistent uninvestigated dyspepsia, in particular with long-term use of PPIs, should undergo gastroscopy with biopsies to rule out the presence of AAG or neoplastic complications (20).

Clinical practice guidelines on the appropriate use of PPIs and the duration of therapy when indicated have been described in several position papers (27, 30–32). Despite this, the misuse of PPIs is common. Taking together our findings and those of the previous papers (3–5, 21–26), clinicians’ awareness of the appropriateness of PPI prescription should be considered as a remarkable topic in daily clinical practice.

Our study suffers from some limitations: data on PPI classes were not recorded, but the GC risk seems not related to the PPI class used (33). The exact dosage of PPIs could also not be reliably collected. This case–control study was conducted at a single center and the sample size was obliged by the available number of AAG patients with GNLs. The serum gastrin values were not available for our AAG patients. Immunohistochemical staining to rule out the neuroendocrine differentiation in our GC series was not carried out; therefore, we are not able to rule out the idea that at least some of the found lesions might be linked to PPI use because of harboring a neuroendocrine differentiation not recognized by conventional immunohistochemistry. Qvigstad et al. (34) demonstrated in a series of GC the presence of neoplastic cells with neuroendocrine and probably ECL cell differentiation, using sensitive immunohistochemical techniques. This point would deserve particular attention, worthy of a further study specifically dedicated.

Nevertheless, our findings seem to add important pieces to the puzzle of the intriguing relationship between pharmacological acid suppression and GC risk, inviting us to reflect on the appropriate PPI use.

In conclusion, PPI use before the diagnosis of AAG appears to considerably increase the risk of subsequent GNL development. Considering the common misuse of PPIs, physicians should regularly reevaluate the appropriateness of ongoing PPI therapy, in particular in patients with a clinical suspicion of or already-diagnosed AAG.

## Data Availability Statement

The original contributions presented in the study are included in the article/**Supplementary Material**. Further inquiries can be directed to the corresponding authors.

## Ethics Statement

The studies involving human participants were reviewed and approved by Sant’andrea Hospital ethical committee. The patients/participants provided their written informed consent to participate in this study.

## Author Contributions

EL and ED planned the study. EL, ED, MB, and MC conducted the study, collected and interpreted data, and drafted the article. ED and GE performed gastroscopies. EL and BA supervised the study. All authors have approved the final draft submitted.

## Conflict of Interest

The authors declare that the research was conducted in the absence of any commercial or financial relationships that could be construed as a potential conflict of interest.

## Publisher’s Note

All claims expressed in this article are solely those of the authors and do not necessarily represent those of their affiliated organizations, or those of the publisher, the editors and the reviewers. Any product that may be evaluated in this article, or claim that may be made by its manufacturer, is not guaranteed or endorsed by the publisher.
