# Supplementary material for: The Impact of Proton Pump Inhibitors on the Development of Gastric Neoplastic Lesions in Patients With Autoimmune Atrophic Gastritis
Source: Front Immunol. 2022 Jul 22;13:910077. doi: 10.3389/fimmu.2022.910077 (PMC9353125; doi:10.3389/fimmu.2022.910077)
Supplement: Supplementary file 1 [file Table_1.pdf]

Supplementary Table 1.

STROBE Statement—Checklist of items that should be included in reports of ***case-control studies***

	Item No	Recommendation	Page No
**Title and abstract**	1	(*a*) Indicate the study's design with a commonly used term in the title or the abstract	2
		(*b*) Provide in the abstract an informative and balanced summary of what was done and what was found	2
**Introduction**			
Background/rationale	2	Explain the scientific background and rationale for the investigation being reported	2
Objectives	3	State specific objectives, including any prespecified hypotheses	2,3
**Methods**			
Study design	4	Present key elements of study design early in the paper	3
Setting	5	Describe the setting, locations, and relevant dates, including periods of recruitment, exposure, follow-up, and data collection	3
Participants	6	(*a*) Give the eligibility criteria, and the sources and methods of case ascertainment and control selection. Give the rationale for the choice of cases and controls	3
		(*b*) For matched studies, give matching criteria and the number of controls per case	3
Variables	7	Clearly define all outcomes, exposures, predictors, potential confounders, and effect modifiers. Give diagnostic criteria, if applicable	3,4
Data sources/ measurement	8	For each variable of interest, give sources of data and details of methods of assessment (measurement). Describe	4

		comparability of assessment methods if there is more than one group	
Bias	9	Describe any efforts to address potential sources of bias	
Study size	10	Explain how the study size was arrived at	3, Figure 1
Quantitative variables	11	Explain how quantitative variables were handled in the analyses. If applicable, describe which groupings were chosen and why	
Statistical methods	12	(*a*) Describe all statistical methods, including those used to control for confounding	4
		(*b*) Describe any methods used to examine subgroups and interactions	4
		(*c*) Explain how missing data were addressed	
		(*d*) If applicable, explain how matching of cases and controls was addressed	3
		(*e*) Describe any sensitivity analyses	4
**Results**			
Participants	13	(a) Report numbers of individuals at each stage of study—eg numbers potentially eligible, examined for eligibility, confirmed eligible, included in the study, completing follow-up, and analysed	4
		(b) Give reasons for non-participation at each stage	
		(c) Consider use of a flow diagram	Figure 1
Descriptive data	14	(a) Give characteristics of study participants (eg demographic, clinical, social) and information on exposures and potential confounders	4,5, table 1, 2, 3
		(b) Indicate number of participants with missing data for each variable of interest	
Outcome data	15	Report numbers in each exposure category, or summary measures of exposure	4,5 table 1, 2, 3
Main results	16	(*a*) Give unadjusted estimates and, if applicable, confounder-adjusted estimates and their precision (eg,	4,5 table 1, 2, 3

	95% confidence interval). Make clear which confounders were adjusted for and why they were included
	(*b*) Report category boundaries when continuous variables were categorized
	(*c*) If relevant, consider translating estimates of relative risk into absolute risk for a meaningful time period

2 Supplementary Table 2.

Main features of patients with autoimmune atrophic gastritis who developed gastric cancer or high-grade dysplasia at follow-up.

ESD: endoscopic submucosal dissection; EMR: endoscopic mucosal resection; TNM: tumor-node-metastasis.

Age, years	Gender	Endoscopically visible lesion	Site	Histology	TNM staging	Treatment	Outcome
**Gastric cancer**							
**50**	Female	0-IIa with demarcation line, 30 mm	Corpus	Intestinal-type adenocarcinoma	pT1B N0 M0	Surgery	Alive
**82**	Male	0-IIa+IIc, 40 cm	Antrum	Intestinal-type adenocarcinoma	pT3 N3 M0	Surgery	Died
**47**	Male	Ulcer-like, 40 cm	Antrum	Intestinal-type adenocarcinoma	pT4 N2 M0	Surgery	Alive
**83**	Female	0-IIa, with demarcation line, 20 mm	Corpus	Intestinal-type adenocarcinoma	pT1 N0 M0	ESD	Alive
**73**	Female	0-IIc+IIa, 30 mm	Antrum	Intestinal-type adenocarcinoma	pT1 N0 M0	Surgery	Alive
**77**	Male	0-IIa+IIc, 20 mm	Corpus	Intestinal-type adenocarcinoma	pT2 N0 M0	Surgery	Alive
**71**	Male	0-IIa+IIc, 30 cm	Angulus	Intestinal-type adenocarcinoma			Lost at follow-up
**71**	Female	0-IIc+IIa, 40 mm	Antrum	Intestinal-type adenocarcinoma	pT2 N2 M0	Surgery	Alive
**78**	Female	0-IIa+IIc, 20 mm	Antrum	Intestinal-type adenocarcinoma	pT1a N0 M0	ESD	Alive
**71**	Female	0-IIa+IIc, 40 mm	Antrum	Intestinal-type adenocarcinoma	pT3 N1 M0	Surgery	Alive
**High-grade dysplasia**							
**77**	Female	0-IIa, 15 mm	Antrum	Intestinal-type dysplasia		Surgery	Died
**75**	Female	0-IIa+IIc, 15 mm	Angulus	Intestinal-type Dysplasia		ESD	Alive
**79**	Male	0-IIa, 30 mm	Angulus	Intestinal-type Dysplasia		ESD	Alive

3 Supplementary Table 3.

Main features of patients with autoimmune atrophic gastritis who developed low-grade dysplasia at follow-up.

ESD: endoscopic submucosal dissection; EMR: endoscopic mucosal resection; GC: gastric cancer.

Age, years	Gender	Endoscopically visible lesion	Site	Treatment	Recurrence	Recurrence (years)	Site of recurrence	Progression to GC	Outcome
**Low-grade dysplasia**									
**49**	Female	0-Is, 7 mm	Antrum	EMR	Yes	7	Antrum (the same)	No	Alive
**71**	Female	0-IIa+IIb, 20 mm	Antrum	ESD	No	-	-	No	Alive
**55**	Female	0-Is, 3 mm	Antrum	EMR	-	-	-	-	Lost at follow-up
**80**	Female	0-Is, 2 mm	Corpus	EMR	No	-	-	No	Alive
**63**	Female	0-Is, 6 mm	Corpus	EMR	Yes	2	Corpus (the same)	No	Alive
**71**	Male	0-Is, 15 mm	Corpus	EMR	No	-	-	No	Alive
**79**	Male	0-Is, 8 mm	Angulus	EMR	No	-	-	-	Alive
**64**	Male	0-Is, 3 mm	Antrum	EMR	-	-	-	-	Lost at follow-up
**57**	Male	0-Is, 3 mm	Antrum	EMR	No	-	-	No	Alive
**69**	Female	0-IIa, 15 mm	Corpus	EMR	No	-	-	No	Alive
**86**	Female	0-Is 7 mm	Corpus	EMR	No	-	-	No	Died
**70**	Female	0-Is, 3 mm	Antrum	EMR	No	-	-	No	Alive
**70**	Male	0-IIb, 4 mm	Antrum	EMR	Yes	4	Antrum (the same)	No	Alive
**61**	Female	0-IIa,	Angulus	EMR	No	-	-	No	Alive

		15 mm							
**70**	Male	0-IIc+IIa, 5 mm	Antrum	EMR	No	-	-	No	Alive
**76**	Male	0-IIa, 15 mm	Antrum	ESD	No	-	-	No	Alive
**83**	Female	0-Is, 8 mm	Antrum	EMR	No	-	-	No	Alive
**78**	Male	0-Is, 10 mm	Corpus	EMR	No	-	-	No	Alive
**74**	Female	0-Is, 4 mm	Antrum	EMR	No	-	-	No	Alive
**44**	Male	Flat area with demarcation-line, 5 mm	Antrum	Biopsies	No	-	-	No	Alive
**60**	Female	0-Is, 10 mm	Corpus	EMR	No	-	-	No	Alive
